# Naming a Structured World: A Cultural Route to Duality of Patterning

**DOI:** 10.1371/journal.pone.0037744

**Published:** 2012-06-19

**Authors:** Francesca Tria, Bruno Galantucci, Vittorio Loreto

**Affiliations:** 1 Complex Systems Lagrange Lab, Institute for Scientific Interchange, Torino, Italy; 2 Department of Psychology, Yeshiva University, New York, New York, United States of America; 3 Haskins Laboratories, New Haven, Connecticut, United States of America; 4 Physics Department, Sapienza Università di Roma, Rome, Italy; University Of Cambridge, United Kingdom

## Abstract

The lexicons of human languages organize their units at two distinct levels. At a first combinatorial level, meaningless forms (typically referred to as phonemes) are combined into meaningful units (typically referred to as morphemes). Thanks to this, many morphemes can be obtained by relatively simple combinations of a small number of phonemes. At a second compositional level of the lexicon, morphemes are composed into larger lexical units, the meaning of which is related to the individual meanings of the composing morphemes. This duality of patterning is not a necessity for lexicons and the question remains wide open regarding how a population of individuals is able to bootstrap such a structure and the evolutionary advantages of its emergence. Here we address this question in the framework of a multi-agents model, where a population of individuals plays simple naming games in a conceptual environment modeled as a graph. We demonstrate that errors in communication as well as a blending repair strategy, which crucially exploits a shared conceptual representation of the environment, are sufficient conditions for the emergence of duality of patterning, that can thus be explained in a pure cultural way. Compositional lexicons turn out to be faster to lead to successful communication than purely combinatorial lexicons, suggesting that meaning played a crucial role in the evolution of language.

## Introduction

Charles Hockett [1], [2] lists thirteen design-features of animal communication, identifying three of them as specific to human language. These are the *displacement* (the possibility to speak about things remote in space or time), *productivity* (the possibility to say things that have never been said before) and the so-called *duality of patterning*. A language exhibits duality of patterning when it is organized at two distinct levels [1], [3], [4]. At a first level, meaningless forms (typically referred to as phonemes) are combined into meaningful units (henceforth this property will be referred to as *combinatoriality*). For example, the English forms/k/, /a/, and/t/are combined in different ways to obtain the three words/kat/, /akt/, and/tak/(respectively written ‘cat’, ‘act’ and ‘tack’). Because the individual forms in them are meaningless, these words have no relation in meaning even though they are made of the same forms. This is a very important property, thanks to which all of the many words of the English lexicon can be obtained by relatively simple combinations of about forty phonemes. It is important to stress that if phonemes had individual meaning, this degree of compactness would not be possible. Human lexicons exhibit a second level of structure, where meaningful units (typically referred to as morphemes) are composed into larger units, the meaning of which is related to the individual meaning of the composing units (henceforth this property will be referred to as compositionality). This higher level of organization, unlike the combinatioral level, includes semantics. For example, the meaning of the word ‘bedroom’ is related to the meaning of the words ‘bed’ and ‘room’ which composed it. From this point of view, it is clear that productivity is not independent of duality of patterning [4], [5]. The compositional level includes syntax as well. For example, the meaning of the sentence ‘cats avoid tacks’ is related to the meaning of the words ‘cats’, ‘avoid’, and ‘tacks’. However, for the sake of simplicity, in this paper we focus exclusively on the lexicon level. In a non-combinatorial lexicon there would be a word composed by unique speech sounds for each meaning in the lexicon. Lexicons of this kind do not exist in natural languages. Indeed, natural language lexicons tend to be highly combinatorial: meanings run in the tens of thousands while speech sounds typically run in the dozens [6]. A lexicon could however be combinatorial but not compositional: this would mean that, for each meaning in the lexicon, there would be a distinct monomorphemic word. In the case of English, for example, a word such as ‘bedroom’ would have to be replaced by a monomorphemic word like ‘attic’. If this were the case, similarities in meaning would very rarely - and only accidentally - correspond to similarities in form. Lexicons of this kind, although possible, do not exist in natural languages and systematic relations between form and meaning are widespread. For example, the process through which the word ‘bedroom’ is created is fairly productive in English (e.g., ‘bathroom’, ‘bedtime’, etc.) as well as in many other languages [7]. Languages vary considerably with respect to the extent to which they manifest compositionality at the lexical level. However, even languages in which compositionality is used the least - such as Classical Chinese [8] - have a great number of multimorphemic words and, in this sense, fully exhibit lexical duality of patterning.

In this paper we focus on the mechanisms that could lead to the establishment of duality of patterning in a lexicon. To be sure, there have been a number of previous attempts to explain the emergence of linguistic units composed by a blending of sub-units [9]–[21]. However, a clear coherent picture is still lacking, most likely because the two levels of duality of patterning have been studied independently from each other (see for instance separate modeling efforts in [22] and [23]). The goal of this study is that of attempting to fill this gap, minimizing, at the same time, the number of assumptions we make, e.g., for the set of elementary symbols to be adopted. In particular, we require combinatoriality to emerge out of a virtually infinite set of symbols. Such set is limited only by means of self-organization of individuals through repeated language games with the only purpose of communicating. This is an important novelty with respect to previous attempts in this direction, where a predefined set of symbols is often used, e.g. [9]. In order to address the problem of the emergence of compositionality, we have to introduce some form of semantics in our model. We use a simple representation of the conceptual space that we model as a graph, where nodes are objects or concepts to be named and links represent any kind of semantic connection between them. It is important to emphasize that we do not rely on any predefined linguistic category or predefined meaning [16]–[18], [24]. Also, It is important to emphasize that our model involves exclusively peer-to-peer negotiations which take place on cultural time-scales in large populations. Often studies in this area have been focused on evolutionary time-scales (e.g., [24], [25]), neglecting such cultural time-scales. In contrast, there is evidence suggesting that humans are capable of evolving languages with duality of patterning in a matter of only one or two generations (consider for instance the Nicaraguan Sign Language [26], [27]).

In order to address these issues we introduce a general modeling framework where the question of the emergence of lexicons featuring duality of patterning is addressed in a self-consistent way. We consider an initially *blank slate* population of individuals committed to bootstrapping a lexicon using an open-ended set of forms in a large meaning space modeled as a graph. This removes altogether any notion of prototype, predefined linguistic category, predefined meaning or relation among objects and symbols. We show in particular that errors in communication as well as a blending repair strategy, sometime adopted by individuals when communication fails, are sufficient conditions for the emergence of compositional as well as combinatiorial structures in the emerging lexicon, demonstrating in this way that duality of patterning can emerge via simple cultural processes.

## Methods

### The Blending Game

The computational model used in this experiment involves a population of *N* artificial agents living in a world composed by *M* objects. The *M* objects are not independent from each other but they share features that establish pairwise links among them. In this sense the objects are intended as a conceptual space [28]–[30] grounded in cognition. A precise and complete description of such a conceptual space being out of reach, we model it as a graph, making very general hypotheses about its structure and checking the robustness of our results with respect to different plausible choices of the graph structure. Also, for simplicity, we consider a population with an homogeneous structure, where each individual has the same probability of interacting with everyone else.

Starting from scratch and without any central coordination, the individuals perform pairwise language games aimed at naming the objects in their world. Each individual is characterized by its inventory or memory, i.e., a collection of lists of name-object associations that are empty at the beginning of the process and evolve dynamically as time progresses. As already introduced in language games [31] devoted to Naming [32]–[34] and Category formation [35]–[37], at each time step two agents are randomly selected, one to play as Speaker (S), the other one as Hearer (H). S randomly chooses an object named Topic (T) to discuss, his goal being that of producing a *word* (see below for a definition) which enables H to correctly identify T. In order to study the emergence of duality of patterning, we consider here the possibility for any word to be composed either of a single form or of an ordered linear concatenation of forms (here a form is intended as the meaningless unit of a signal). The emergence of words composed of more than one form arises in a natural way, according to the following mechanism.

As stated above, S has to name a topic T. If S does not have already a word for it, she invents a word consisting of a single form which is novel for the entire population. The constraint for the new word to consist of a new form for the whole population corresponds to a virtually infinite, i.e., open-ended, repertoire of symbols. We will see that even in this extreme scenario, only a very limited number of different forms gets eventually fixed in the population’s lexicon. If S already possesses one or more words for T in its inventory, she chooses the last winning-word, if it exists (i.e., the word that allowed the last successful communication about T), or a random word otherwise.

**Figure 1:**
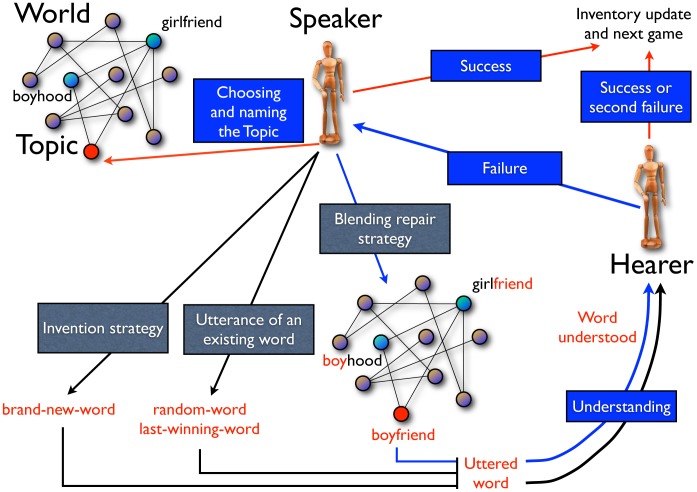
Diagram of the Blending Game. In each game a Speaker and a Hearer are chosen. The Speaker chooses a Topic and utters a name for it (i.e., it produces one or more meaningless forms, consisting of unique identifiers, e.g., 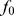, 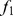, etc.). If her inventory for the Topic is empty she invents a brand new word, i.e., a word never appeared in the whole population for all the possible objects. Alternatively, she chooses one of the existing word (the last-winning one). The hearer has to understand the uttered word and to relate it to an object on the basis of the content of her previously recorded information (her repertoire). The understanding phase is ruled by the parameter 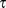, a characteristic memory scale introduced through eq. 1. If the communication fails the Speaker adopts a blending repair strategy which consists in inventing a new word composed by reusing parts of already proposed words, preferably associated with object nearest neighbors of the chosen topic. In the example depicted, *boyfriend* is composed out of *boyhood* and *girlfriend*. If also after the adoption of the repair strategy the communication fails, the Hearer updates her inventory with the word just heard. If the game is a success, the inventories of both the Speaker and the Hearer for the Topic are cleared except for the successful word.

Upon hearing the word from S, H tries to guess T. The guessing procedure consists of two parts. First, H has to understand the word uttered by S, second she has to associate an object to that. As an essential ingredient of the model, we consider an initial imperfect understanding due to noise in communication. More specifically, the first time H hears a form, she likely misunderstands it, while after successive encounters of the same form, she will eventually understand it. In this way a finite fidelity in the message transmission is introduced such that H parses the heard word into its component forms and correctly understands each form with a time dependent probability:
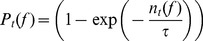
(1)where 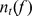 is the number of times H has heard the form *f* up to time *t* and 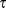 is a characteristic memory scale. If H does not understand a form, she replaces it with a random form from her inventory, i.e., a random form among all those composing any word she used to name any object. If her inventory is still empty, she replaces the form not understood with a brand new one, i.e., an invented form. The word understood by H is thus composed by the same number of forms as the uttered word and it is equal to the uttered word except, possibly, for the misunderstood forms. Finally, H checks whether she has already the word understood in her inventory, associated to any object. If so, H selects the corresponding object and if the word is associated with more than one object she chooses at random among them. If H correctly guesses T, the game is a *success* and both agents delete all but the winning word from their inventories for T. If this is not the case, the game is a *failure* and H adds the word understood to her inventory for T (see Figure 1 in the Information S1).

Overall, the mechanism described here places an intrinsic limit on the number of distinct forms eventually used by the agents [22]. We will see that this phenomenon enhances the probability that, during the negotiation process, the inventory of an individual contains two or more objects with a common associated word. The appearance of this *Homonymy* (see below for a quantitative definition) turns out to be only a transient phenomenon, and the homonymy eventually disappears as a result of pairwise interactions aiming at a successful communication.

Further, in case of failure, S has a second (and last for the current interaction) chance to communicate about T, this time adopting a *blending repair strategy* (see for instance [5] for the literature about conceptual blending). In particular, S chooses two objects in the world. If T has at least two neighbors with non-empty inventories for S, she chooses among them, otherwise she considers two random objects. Having chosen the two objects, S takes a word from each of them and composes the new word for T by taking the initial part (of random arbitrary length) from one and the final part (again of random arbitrary length) from the other, keeping the order of the parent words in the composed word. When the blending repair strategy is adopted, the same communication and understanding procedure as before is considered and the game is declared a *success* or a *failure* with the same criteria as in the first communication attempt. Thus, in case of success both agents delete all their words from their inventories relative to T, apart from the winning one. In case of failure H inserts also this second word, as she understands it, in its inventory for the topic T. Figure 1 synthetically summarizes the structure of the game and gives an example of the blending repair strategy.

In order to model the conceptual space, we considered two different structures: Erdős -Rényi (ER) random graphs [38], [39] and uncorrelated random scale-free (UCM) graphs [40]. However, since the behaviour of the system does not depend on the particular structure of the conceptual space (both from a qualitative and quantitative point of view), we report here the results obtained with ER random graphs; results obtained with UCM are reported in the Information S1 (SI). All the results that follow are averages over different (100) realizations of the process on the same conceptual graph. We checked that a further average on different graphs with the same statistical properties (e.g., ER random graphs with the same link probability 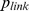) produces fluctuations that remain within the reported error bars.

In summary, the main ingredients of the Blending Game are two. First, there is noise in comprehension and this applies to all games whenever a hearer tries to understands a word uttered by a speaker. This is an essential ingredient responsible for keeping the number of different forms shared by the whole population limited and low, without any a priori constraints on it. Second, along with the basic strategy of word creation, the game features a repair strategy that exploits the structure of the world to create new words. Sometime the blending is independent from the meaning of these words, feeding the combinatorial level of the lexicon. On other occasions, the blending involves words which are taken from the inventories of related objects (that is, objects connected by a link to the current topic), thus feeding the compositional level of the lexicon. Thanks to this compositional blending, part of the semantic structure which organizes the objects in the world percolates into the lexicon.

Our aim now is that of investigating under which conditions duality of patterning emerges in a population of individuals and what are the conditions leading to the emergence of combinatoriality as well as of compositionality. It is important to notice that, while duality of patterning is an emergent properties of language at the population level, the rules of the blending game reflects basic cognitive abilities of individuals and as such they are not conceived to bias the outcome of the evolution towards duality of patterning. Indeed, purely non-combinatorial or combinatorial but non-compositional lexicons can always emerge, as we shall detail in the next section.

## Results

**Figure 2:**
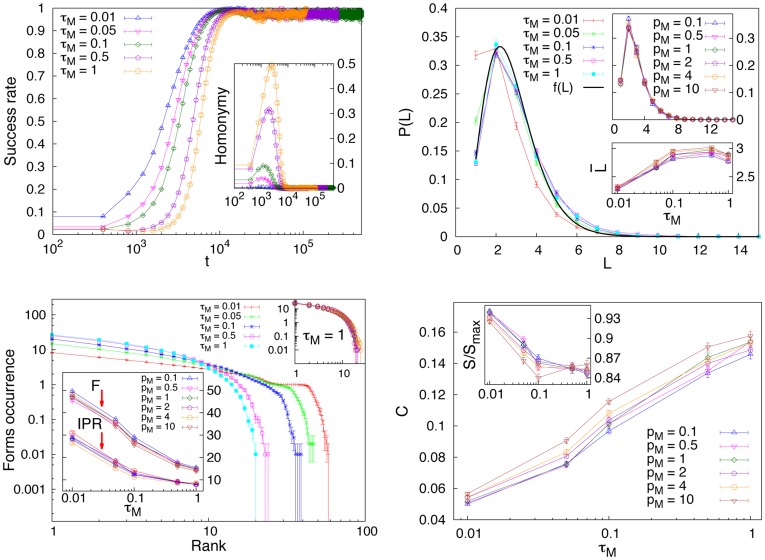
Dynamics and statistical properties of the emerging lexicon. **Top Left.** *Success rate and homonymy as a function of time.* Success rate and homonymy are reported as a function of time (number of games), averaged over sliding time windows. Homonymy is here defined as the number of pairs of objects that have at least an associated word in common in an individual inventory, divided by the number of pairs 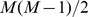 and averaged over all the agents. We define here a normalized link probability for the ER random graph as 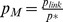, where 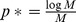 is the threshold in order to have, with probability one in the infinite size limit (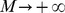), a giant connected component equal to the whole graph. Similarly, a normalized time scale parameter is defined as 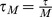. Results are reported for 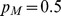 and for different values of the learning parameter 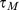. **Top Right.** *Word length distribution.* In the main figure the distribution of word length *L* for different 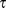 values is reported, again fixing 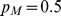. 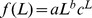 is the observed empirical function [41] (fitting parameters 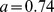, 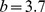, 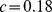). In the top inset we show the same distribution fixing 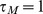 for different values of 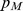. Note that here the curves overlap, indicating that the word length distribution does not depend on the objects graph connectivity. In the bottom inset the *average word length*, *L*, as a function of 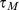 is reported, for different values of 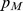. **Bottom Left.** *Frequency-rank distribution for elementary forms*. In the main figure the frequency-rank distribution for elementary forms is shown for different values of the parameter 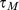 keeping fixed 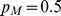. In the top inset we show the same distribution fixing 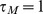 and for different 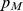, showing that the distribution of elementary forms again does not depend on the objects graph connectivity. In the bottom inset both the *number of distinct elementary forms* composing the lexicon and the *effective number of distinct elementary forms*, as defined in the text, are reported, as a function of 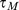 and for different values of 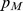. **Bottom Right.** *Combinatoriality*. Combinatoriality *C* (see the text for definition) for different values of 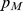 as a function of 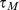. In the inset the *normalized entropy*, as defined in the text, is reported, again for different values of 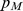 as a function of 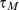. In all the measures reported above the number of agents and the number of objects in the environment are fixed respectively to 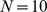 and 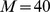 and results are averaged over 100 realizations of the dynamics on the same objects graph (see main text for further details).

We report here the results for a fixed value of the population size *N* and of the number of objects in the environment *M*. The dependence of the results on *N* and *M* is reported in the Information S1. We recall that all the individuals start with empty inventories and no preexisting knowledge. As time goes on, the inventories are progressively populated with words either invented (from scratch or through the blending repair strategy) or heard from other individuals (possibly misunderstood). The first important observation is that the dynamics always leads the system to an asymptotic state in which all the individuals in the population share the same linguistic repertoire, each object being named by a single distinct name (e.g., 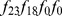 is a typical word composed by the elementary forms 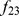, 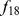 and 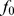). This means that no synonymy nor homonymy are present in the asymptotic state. Figure 2 (top left) illustrates how the communicative success starts from zero and progressively increases, leading eventually to a fully successful shared communication system with an “S”-shaped time behaviour. At the same time, homonymy is defeated after a transient phase in which it spreads widely (see inset of the top-left panel of Figure 2).

We now analyze more in details the structure of the emerging lexicon, focusing in particular on its statistical signatures and introducing suitable indicators to quantify combinatoriality and compositionality.

### Lexicon Statistics

The first quantity we look at is the word length distribution. Here the length is measured in terms of the forms each word is composed of. Figure 2 (top right) shows the word length distribution for different values of the normalized memory parameter 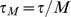 and of the normalized graph connectivity 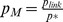, with 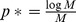 (see caption of Figure 2 for the rationale behind this definition). While the word length distribution is not affected by the graph connectivity, a very light dependence on 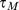 is observed. It is remarkable that the observed distribution is quantitatively similar to the one observed empirically [41] (Figure 2, top right, displays also the empirical fitting function). The average word length remains bounded around three forms (bottom inset of Figure 2, top right). This limitation on the word length is an outcome of the negotiation dynamics, emerging without any explicit constraint on it.

As a further step to investigate the properties of the emerged lexicon, we turn our attention to the frequency distribution of the different forms composing the words (Figure 2, bottom left). As in the case of the word length distribution, the frequency-rank distribution for forms does not depend on 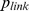 (see top inset of bottom-left panel of Figure 2), while it shows a clear dependence on the memory parameter 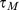. In particular, the higher 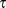 (for *M* fixed), i.e., the lower the fidelity in communication, the smaller the number of distinct forms on which the agents eventually find agreement. Since the invention rate of new forms does not depend on 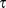, the effect of a high 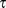 is that of strengthening the selection process, reducing in this way the number of forms that get fixed in the population. It is worth noticing that, for large values of 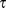, the frequency-rank distribution we observe is remarkably similar to the corresponding distribution observed in human languages [42] for which a Yule-like distribution [43] has been hypothesized (see Information S1). In addition, also the number of distinct forms (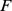) as well as its effective number, as measured by the inverse participation ratio (IPR) (see bottom left inset of Figure 2 (bottom left)) are strikingly similar to the corresponding values in human languages. We recall that the IPR is defined as 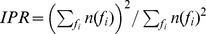, where 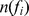 is the number of times 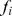 is used in the whole emerging lexicon, and reaches its maximum value *F* when all the forms are equally used. A complementary measure of the statistics of use of the different forms in the lexicon is given by the entropy. The entropy of the elementary forms distribution is defined as 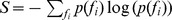 where 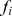 is the generic elementary form and 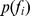 is the frequency of occurrence of 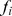 in the whole emerging lexicon estimated as 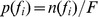. The inset of Figure 2 (bottom right) reports the normalized entropy, i.e., the entropy divided by its maximum value for the actual number *F* of distinct forms, 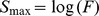, reached when all the forms are equiprobable. All these measures demonstrate that the stronger is the noise in comprehension the smaller is the number of distinct forms fixed in the population and the more ordered is the distribution of their frequencies.

### Emergence of Combinatoriality

We are now ready to introduce a measure of combinatoriality to quantify the property of a communication system to combine and re-use a small number of elementary forms to produce a large number of words. Following the idea in [44], we introduce a real-valued quantity ranging in the interval [0∶1] that quantifies how frequently forms recur in the emerged lexicon according to the following formula:
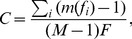
(2)where the sum runs over all the *F* distinct forms present in the emerged lexicon and 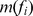 is the number of distinct objects whose name includes the form 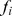. The term 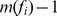 takes into account only the forms that are used to name at least two objects, i.e., only the forms that are actually re-used. *M* is again the number of objects to be named. The results for the combinatoriality are reported in Figure 2 (bottom right), as a function of 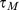 and for different values of 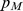. Again, a negligible dependence on 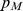 is found, while maximal combinatoriality (and minimal entropy) occurs for high values of 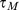. At the same time, the limit of small 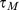 corresponds to small values of combinatoriality. This can be understood if one thinks that for a perfect level of understanding there is no selective pressure acting on the different forms and many distinct forms are eventually fixed in the lexicon with a small re-use rate, i.e., little combinatoriality. In a sense, the limit of small 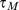 is the *holistic* limit of our model, i.e., a limit in which forms stand for the meaning as a whole and have no meaningful subparts. In our case the word holistic refers both to the case where every word of the lexicon is composed by a single form or cases where several forms are needed for a word but none of them is re-used in the whole lexicon. We again refer to the Information S1 for further details concerning the dependence of the results on *N* and *M*.

Summarizing, when the constraints on understanding and learning new forms are sufficiently strong, one finds, at the lexical level, features similar to the ones observed in real languages such as the word length distribution and the number and the frequency of use of the different forms. At the same time, combinatoriality emerges as a workaround to overcome the problem of noisy communication.

### Emergence of Compositionality

Let us now turn to the compositional aspects of the lexicon, the aim here being that of establishing whether, in the emerged lexicon, words for semantically related concept are expressed through morphologically similar words.

Here we measure the semantic similarity of two objects in terms of their distance on the graph describing the conceptual environment. In addition we need to define a measure of morphological similarity between words. To this end we introduce a Master-Mind-like (MM) measure and we refer to the Information S1 for the results obtained with other similarity measures.

Given two words 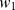 and 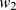, each composed of a certain number of forms, the Master-Mind-like (MM) measure of form similarity is defined as follows: after the two words have been aligned, either making the left-end or the right-end coincide, we sum 1 whenever the two words share the same form in the same position and 0.5 for the same form in different positions. The MM measure will be the maximum between the left-end and the right-end alignments. The MM measure conveys the idea that meaningful forms are often included in words as a suffix or a prefix, and in general in a well defined position.

**Figure 3:**
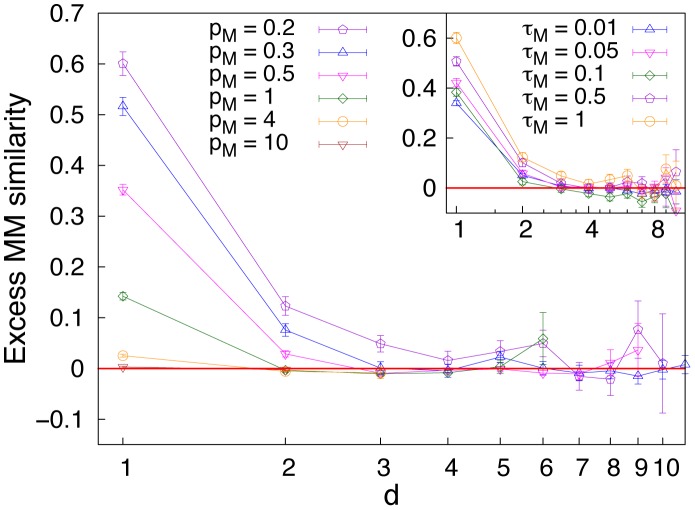
Excess Master-Mind-like similarity of words as a function of the distance *d* of the corresponding objects on the graph. The difference between the actual similarity and its random value computed on a reshuffled communication system is shown (see text for details). A decrease in the excess similarity as a function of the topological distance *d* is the signature of the emergence of compositionality; in particular, compositionality implies higher similarity among words which are closer in the semantic space. The topological distance on the object graph is our proxy for the semantic relatedness. The MM similarity (see text) is adopted here to compute similarity between words. The results are reported for 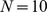 and 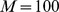. Results are shown for different values of the objects graph connectivity 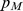, keeping fixed 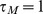 (main figure) and for different values of 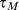 keeping fixed 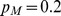 (inset).

As a measure of compositionality, we measure the *excess similarity* of words used to name related objects (at low distance in the graph) when compared to the similarity of randomly chosen words. In order to do that, we consider the average difference between the similarity between each pair of words as a function of the distance of the corresponding objects in the conceptual graph, and the same value computed in the random case, obtained by reshuffling the association between words and objects. In the main graph of Figure 3 we report the excess similarity for a fixed value of 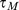 and several values of 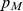 as a function of the topological distance *d* on the conceptual graph. The inset reports the same measure for a fixed value of 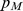 and different values of 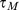. Compositionality is evident in the figure: the more closely related the words, the higher the excess similarity. Moreover, the excess similarity only weakly depends on 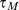, while strongly depends on 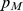. This indicates that a percolation of the organization of the world into the lexicon is possible when the world has a non-trivial semantic structure, i.e., in our case when 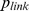 is different from zero and from one. In the former case no relation between objects exists, while in the latter case all the objects are equally related (all are at distance one in the graph). Diluted graphs are more prone to induce compositionality. In contrast, the enhancement of the compositional structure due to an increase of 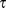 is quite mild.

**Figure 4:**
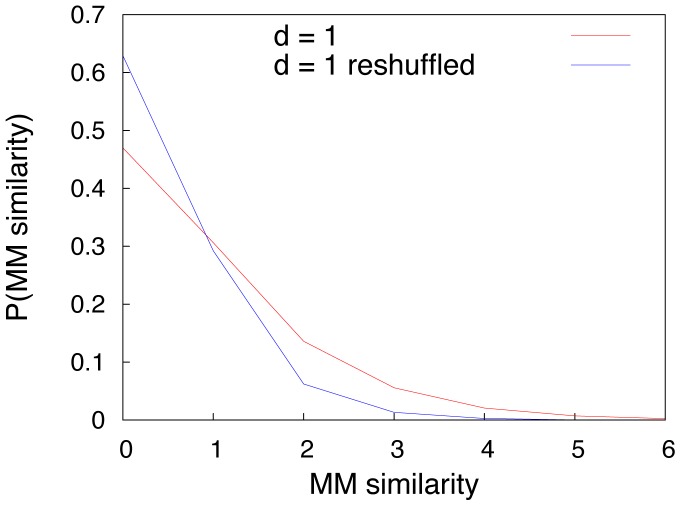
Histogram of the Master-Mind-like similarity of words used to name objects at distance 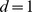 in the conceptual graph. A comparison between the histograms of the MM similarity of words used to name objects at distance 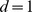 in the conceptual graph and in the reshuffled graph is reported. Note that the histogram corresponding to the original graph is biased towards higher values of similarity with respect to the reshuffled case, indicating excess similarity for semantically related words. However, both histograms are peaked at zero similarity, indicating that words can be semantically related without sharing similarity in form. Further, this latter case concerns the majority of the words, as is the case with real languages. The reported results are averages over 100 realizations with parameters values 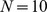, 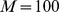, 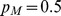 and 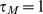.

An important conclusion is that the graph connectivity strongly affects the compositionality of the lexicon, while noise in communication strongly affects the combinatoriality of the lexicon. It is important to stress that the excess similarity featured in the emerging lexicon is comparable to the one observed in real languages, where relations between form and meaning are statistically relevant but of course they are not the rule. Many related objects or concepts, in fact, are expressed by non related forms. In order to clarify this concept, we show in Figure 4 the histogram of the MM similarity between words used in the emerged lexicon to name objects at distance one in the conceptual graph, compared to the corresponding histogram in the reshuffled graph. We note that, even though the excess similarity with respect to the reshuffled case is evident, both histograms peak at similarity zero, meaning that the majority of words used to name related objects do not show any similarity, as it is the case in real languages.

**Figure 5:**
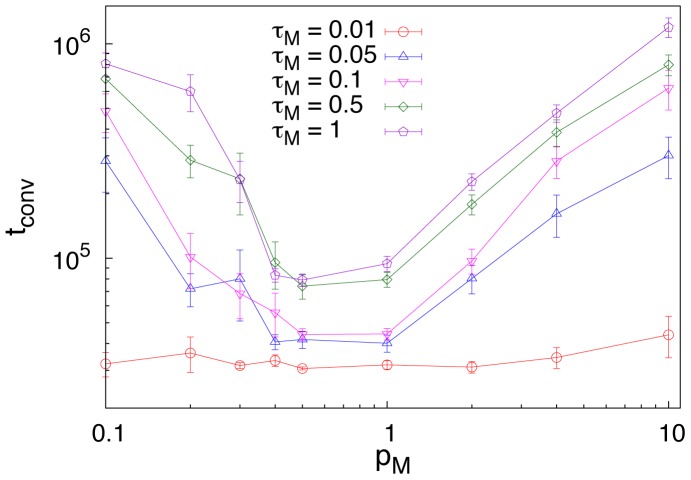
Convergence time. Time needed for the the population of individuals to bootstrap a shared lexicon. All the results are presented for 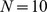 and 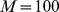 and for several values of 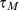. For each value of 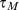, the convergence time has a minimum around the value of the connectivity 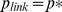 (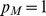). We note that for very small values of 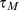, i.e., high fidelity in message transmission, the convergence time is not affected by the structure of the graph since agents barely rely on the blending repair strategy and the naming of each object becomes almost independent of the others.

A last result deserves attention. In Figure 5 we show the *convergence time*, i.e., the time required to a population of individuals to reach agreement, for different values of 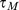, as a function of 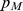. Not surprisingly, higher values of the noise parameter lead to longer times to reach convergence. An interesting result is that convergence time, for 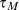 fixed, presents a minimum when the conceptual space has a highly marked structure, that is when the link connectivity is close to the threshold 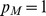, i.e., 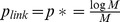, for which the probability of having disconnected components goes to zero. This finding indicates that, if one focuses on the time employed by a population of individuals to bootstrap a successful communication system, an optimal strategy could emerge as the outcome of a complex interplay between the structure of the conceptual space and the compositional strategies adopted by humans.

## Discussion

Through an extensive set of simulations we have shown that a basic language game [33], [35], [36], with the addition of a constraint on the fidelity of message transmission and a minimal semantic mechanism for word coinage, leads to lexicons that exhibit duality of patterning. No predefined relations between objects/meanings and forms are hypothesized and we adopt a virtually infinite, i.e., open-ended, repertoire of forms. Despite this freedom, the number of different forms that get eventually fixed in the population’s lexicon is kept limited by the constraint on transmission fidelity. New words in the lexicon can be created in two ways. They can be holistically introduced as brand new forms or constructed through a blending strategy that combines and re-uses forms taken from other object’s names. At the individual level, the mechanism of blending is thus introduced as an option to be exploited when the first communication attempt resulted in a failure. We note that a blending strategy exists in natural languages (e.g., SMOG = SMoke+fOG). The blending strategy we refer to here, however must be thought as a general mechanism by which different bits of words are put together through blends, compounds or other morphological structures. Interestingly, endowing individuals with an optional blending ability is not sufficient in order to observe a lexicon featuring duality of patterning. We stress again that in the present model we only consider compositionality in lexicon. In order to properly include syntax, we would need to endow the conceptual space with a more sophisticated structure, where nodes could represent nouns/concepts and links could encode semantic relations or specific actions and roles. From this perspective, the present work can be thought as a first step of investigation on the emergence of duality of patterning through a pure cultural way. A next step could focus on investigating how a population of individuals can bootstrap a language where categorization and syntax both emerge as a pure results of communication efforts.

Two crucial manipulations in the game were (i) the degree of transmission fidelity and (ii) the density of the network representing semantic relations among the objects. Combinatoriality, meant as both forms reusing and economy, is only found when the transmission fidelity is sufficiently low. With a high degree of understanding, the number of distinct forms composing the emerged lexicon turns out to be high with respect to the number of objects to be named (in particular, higher that the number of objects), and the resulting lexicon features an extremely low level of combinatoriality (see both figure 2 and the SI). Conversely, an high degree of noise leads to an high level of compactness and combinatoriality. These results suggest that combinatoriality enhances message transmission in noisy environments [22] and emerges as a result of the need of communicative success. In contrast, the level of compositionality is not strongly affected by the level of noise, but strongly depends on how much the conceptual space is structured. In particular, the lexicons developed by the agents exhibited clear signs of compositionality when the networks representing semantic relations among the objects were neither too sparse nor too dense. This can be understood as follows: compositionality does emerge if we are able on the one hand to find common features in different objects, on the other hand to make distinctions so that not all the objects are equally related to each other. Thus, compositionality emerges as a consequence of the organization of our conceptual space [29], [45]. As further analysis, we manipulated the type of semantic network as well as the number of objects and agents in the simulations. Analyses of the converged lexicons revealed two additional significative results. First, the time for lexicons exhibiting compositionality to emerge features a minimum reflecting the outcome of a complex interplay between the structure of the conceptual space and the compositional strategies adopted by humans. Second, none of the results listed above critically depended on the type of semantic network (provided that it has a sufficient degree of structure), the number of objects or the number of agents in the simulations.

These results are important because they demonstrate for the first time that the two sides of duality of patterning can emerge simultaneously as a consequence of purely cultural dynamics in a simulated environment which contains meaningful relations. In addition, the relevance of the interplay between the structure of the conceptual space and simple communication strategies of humans has been highlighted. To our knowledge, this is the first computational result suggesting such dependence. Considering that duality of patterning is a core property of human natural language, the languages we observed in the simulations had a significant element of similarity with human natural language. Additionally, the study provided a number of measures which capture basic linguistic properties of the emerged languages. In other words, the cultural modeling of language emergence has begun to produce predictions which are amenable to testing with realistic samples of natural languages [46], [47]. A further crucial step that we wish to approach in the future is of course that of including syntax as well.

## Supporting Information

Information S1 (PDF)
