# Supplementary material for: Naming a Structured World: A Cultural Route to Duality of Patterning
Source: PLoS One. 2012 Jun 19;7(6):e37744. doi: 10.1371/journal.pone.0037744 (PMC3378539; doi:10.1371/journal.pone.0037744)
Supplement: Information S1 — (PDF) [file pone.0037744.s001.pdf]

Supporting Information for

Naming a structured world: a cultural route to duality of patterning

by Tria et al.

1 Rules of the game

Figure S1 illustrates two examples of a failed and a successful game, respectively.

2 Scaling with the size M of the conceptual space

In Figure S2 we report the analysis of the dependence of the observables considered in the main text on the number M of objects that compose the conceptual space of the agents. We found that the properties related to the emerged lexicon only mildly depend on M. This is important since it allows a quantitative comparison of the model results with the actual human reality, for which in general only a rough estimate of the size of the conceptual space is possible.

3 Scaling with the population size

In Figure S3 we report the analysis of the dependence of the observables considered in the main text on the population size N. Again, we found that the properties related to the emerged lexicon only mildly depend on N.

4 Compositionality with other similarity measures

We consider here two possible variants of the MM similarity adopted in the main text: the *same pairs similarity* (SP) and the *all pairs similarity* (AP) . The SP similarity is defined as follows: given two words w_1 and w_2, we aligned them either making the left-end or the right-end coincide. We then add 1 only when the two words share the same form in the same position. Forms shared by the two words in different positions do not contribute to the SP similarity score. The SP measure will be the maximum between the left-end and the right-end alignments. The AP similarity does not require alignment: given two words w_1 and w_2, a score of 1 is given for each form shared by two words, irrespective of their relative position in those words.

Figure S4 reports the results for the excess similarity (as defined in the main text) when using the SP (left) and the AP (right) similarity scores.

5 Uncorrelated scale-free networks

We have considered networks with different topologies in order to test the robustness of our simulations with respect to the structure of the underlying network. Each node i of a network is first characterized by its degree k_i (number of links) and a first characterization of the network properties is obtained by the statistical distributions of the nodes' degree, $P(k)$. In order to quantify the topological correlations in a network, two main quantities are customarily measured. The clustering coefficient c_i of a node i measures the local cohesiveness around this node [1]. It is defined as the ratio of the number of links between the k_i neighbors of i and the maximum number of such links, $k_i(k_i - 1)/2$. The clustering spectrum measures the average clustering coefficient of nodes of degree k, according to

$$C(k) = \frac{1}{N_k} \sum_i \delta_{k,k_i} c_i \, . \tag{1}$$

Moreover, correlations between the degrees of neighboring nodes are conveniently measured by the average nearest neighbors degree of a vertex i, $k_{nn,i} = \frac{1}{k_i} \sum_{j \in \mathcal{V}(i)} k_j$, and the average degree of the nearest neighbors, $k_{nn}(k)$, for vertices of degree k [2]

$$k_{nn}(k) = \frac{1}{N_k} \sum_i \delta_{k,k_i} k_{nn,i}. \tag{2}$$

In the absence of correlations between degrees of neighboring vertices, $k_{nn}(k)$ is a constant. An increasing behavior of $k_{nn}(k)$ corresponds to the fact that vertices with high degree have a larger probability of being connected with large degree vertices (assortative mixing). On the contrary, a decreasing behavior of $k_{nn}(k)$ defines a disassortative mixing, in the sense that high degree vertices have a majority of neighbors with low degree, while the opposite holds for low degree vertices [3].

In the main text we considered the homogeneous Erdős -Rényi random graph [4,5], in which nodes are linked with a uniform probability p_{link}. In this case, a small diameter and a small clustering coefficient are obtained, and the degree distribution is homogeneous and binomial. The specific properties of the graph depend on p_{link}. In particular if M is the number of nodes, for $p_{link} > log(M)/M$ the graph will almost surely be connected.

We consider here the random scale-free network obtained from the uncorrelated configuration model [6]. It has a broad degree distribution $P(k) \sim k^{-\gamma}$ and it is constructed in such a way to avoid two- and three-vertex correlations, as measured by the average degree of the nearest neighbors $k_{nn}(k)$ and the clustering coefficient of the vertices of degree k, respectively. The average degree distribution is finite for the values of the exponent $2 < \gamma$, and the second moment of the distribution in finite for $3 < \gamma$. We consider here two values for the degree distribution exponent, one below and one above the latter threshold: $\gamma = 2.5$ and $\gamma = 3.5$. We find that all the considered observables do not depend on the value of γ and, moreover, show the same qualitative (and in most cases quantitative) behaviour as when considering an homogeneous Erdős -Rényi random graph with the same number M of nodes (see Figure S5).

References

1. Watts DJ, Strogatz SH (1998) Collective dynamics of 'small-world' networks. Nature 393: 440–442.

2. Pastor-Satorras R, Vázquez A, Vespignani A (2001) Dynamical and correlation properties of the Internet. Physical Review Letters 87: 258701.

3. Newman MEJ (2002) Assortative Mixing in Networks. Physical Review Letters 89: 208701+.

4. Erdös P, Rényi A (1959) On random graphs i. Publ Math Debrecen 6: 290.

5. Erdös P, Rényi A (1960) On the evolution of random graphs. Publ Math Inst Hung Acad Sci 7: 17.

6. Catanzaro M, Boguna M, Pastor-Satorras R (2005) Generation of uncorrelated random scale-free networks. Physical Review E (Statistical, Nonlinear, and Soft Matter Physics) 71: 027103.

7. Tambovtsev Y, Martindale C (2007) Phoneme frequencies follow a yule distribution. SKASE Journal of Theoretical Linguistics 4: 1–11.

8. Yule GU (1944) The statistical study of literary vocabulary. Cambridge University Press.

9. Baronchelli A, Felici M, Caglioti E, Loreto V, Steels L (2006) Sharp transition towards shared vocabularies in multi-agent systems. Journal of Statistical Mechanics P06014.

FAILURE

Before

Speaker

Topic	O_2	O_3	O_4
f1f2f3	f3f3f5	f8f6f1	f9f4f5
f2f7	f1f4f12	f12f3f7	f7f5f7
**f1f1**		f5f9	f3f9
		f3f8f15	

Hearer

Topic	O_2	O_3	O_4
f3f3f12	f4f19f13	f4f19	f73
f17f26f20	f16f47	f15	f20f3
	f23f18f0f0		f27f1f8
	f0f22f0		

After

Speaker

Topic	O_2	O_3	O_4
f1f2f3	f3f3f5	f8f6f1	f9f4f5
f2f7	f1f4f12	f12f3f7	f7f5f7
**f1f1**		f5f9	f3f9
		f3f8f15	

Hearer

Topic	O_2	O_3	O_4
f3f3f12	f4f19f13	f4f19	f73
f17f26f20	f16f47	f15	f20f3
**f1f1**	f23f18f0f0		f27f1f8
	f0f22f0		

SUCCESS

Before

Speaker

Topic	O_2	O_3	O_4
f1f2f3	f3f3f5	f8f6f1	f9f4f5
f2f7	f1f4f12	f12f3f7	f7f5f7
**f1f1**		f5f9	f3f9
		f3f8f15	

Hearer

Topic	O_2	O_3	O_4
f3f3f12	f4f19f13	f4f19	f73
f17f26f20	f16f47	f15	f20f3
**f1f1**	f23f18f0f0		f27f1f8
	f0f22f0		

After

Speaker

Topic	O_2	O_3	O_4
**f1f1**	f3f3f5	f8f6f1	f9f4f5
	f1f4f12	f12f3f7	f7f5f7
		f5f9	f3f9
		f3f8f15	

Hearer

Topic	O_2	O_3	O_4
**f1f1**	f4f19f13	f4f19	f73
	f16f47	f15	f20f3
	f23f18f0f0		f27f1f8
	f0f22f0		

Figure S1. Examples of Games. Top. *Example of a failed game.* In this game the Speaker S selects the Topic and decides to utter the word *f1f1*. This word is unknown to the Hearer H since *f1f1* is not present in any of H's inventories. In this case the game is a failure and H adds the word *f1f1* to her inventory for the Topic. **Bottom.** *Example of a successful game.* In this game the Speaker S selects the Topic and decides to ls utter the word *f1f1*. This word is known to the Hearer H since *f1f1* is present in H's inventory for the Topic. In this case the game is a success and both S and H remove from the Topic's inventory all the competing words but *f1f1*.

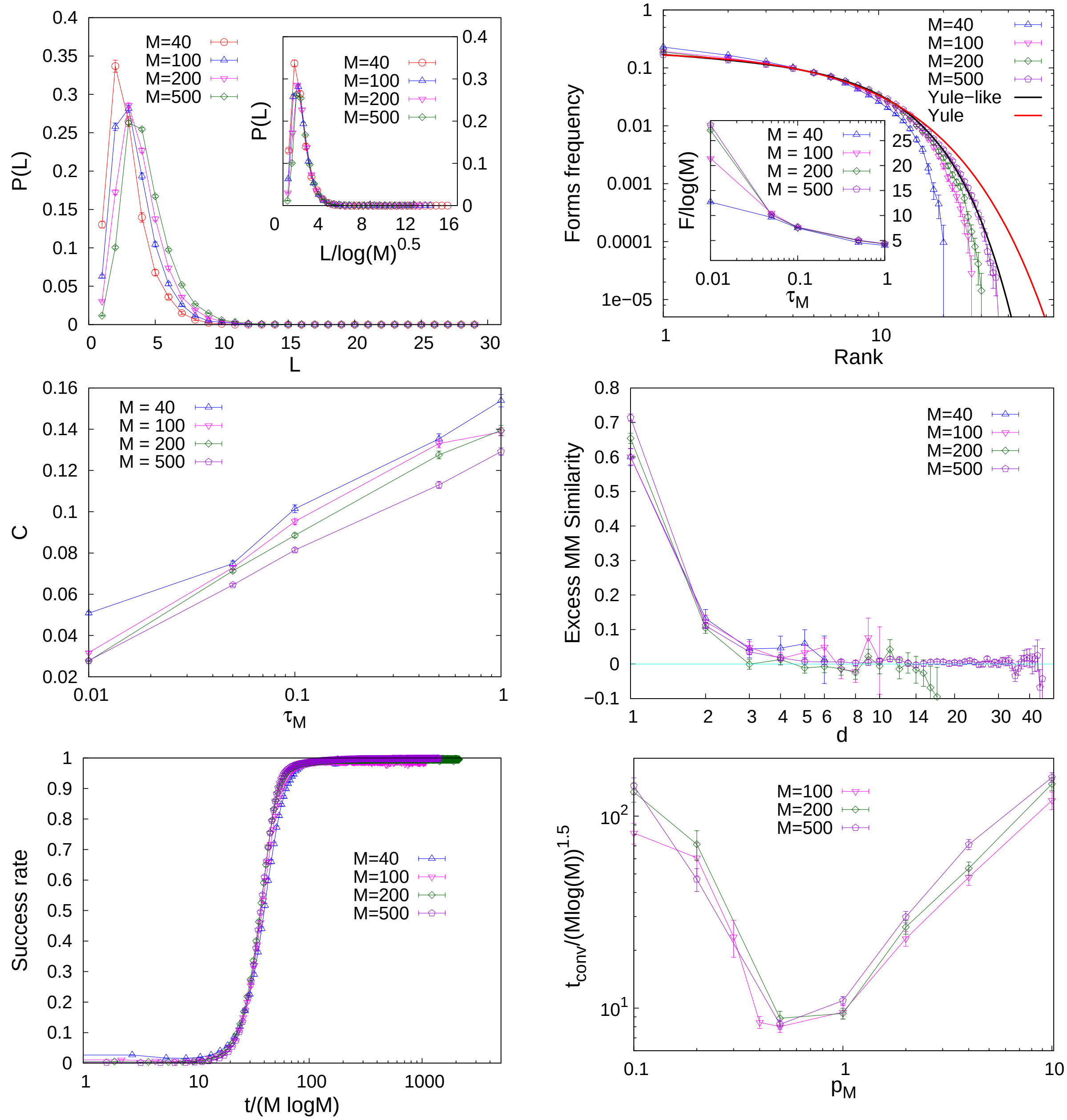

Figure S2. Dependence on M. Top Left. *Word length distribution.* In the main figure the distribution of word length L for different values of M, with $p_M = 1$ and $\tau_M = 1$ is reported. In the inset we report how one obtains the same distributions by rescaling the length by $\log(M)^{0.5}$. This means that there is a very weak dependence of the average word-length on the size of the conceptual space. **Top Right.** *Frequency-rank distribution for elementary forms.* In the main figure we show the frequency-rank distribution for elementary forms for different values of M, again with $p_M = 1$ and $\tau_M = 1$. In the inset we show the number of distinct forms in the emerged lexicons as a function of τ_M and for $p_M = 1$. The curves are rescaled in order to overlap for high value of τ_M. When τ_M is not high enough, the noise ceases to be relevant and both the absolute number of distinct forms increases and the dependence on M becomes stronger. **Center Left.** *Combinatoriality.* Combinatoriality C for different values of M as a function of τ_M and for $p_M = 0.5$. Although the curves are very noisy, the value of the combinatoriality (measured as explained in the main text) is very weakly dependent on M. **Center Right.** *Excess similarity of words as a function of the distance of the corresponding objects on the graph.* The difference between the actual similarity and its random value computed on a reshuffled communication system is shown (see text for details) for different values of M and for $\tau_M = 1$ and $p_M = 0.2$. Again, the results depend very weakly on M and the excess similarity becomes more pronounced as M increases. **Bottom Left.** *Success rate as a function of time.* The success rate is reported as a function of time (number of games) for different values of M, with $p_M = 1$ and $\tau_M = 1$. The curves for different values of M collapse if time is rescaled with a factor $M \log M$. **Bottom Right.** *Convergence time.* Time needed for the system to reach convergence for different values of M, as a function of of p_M and with $\tau_M = 1$. We rescaled the curves in order to superimpose the curves for the value of p_M where the convergence time is minimal. We note that the scaling with M of the convergence time is different from the scaling with M of the success rate.

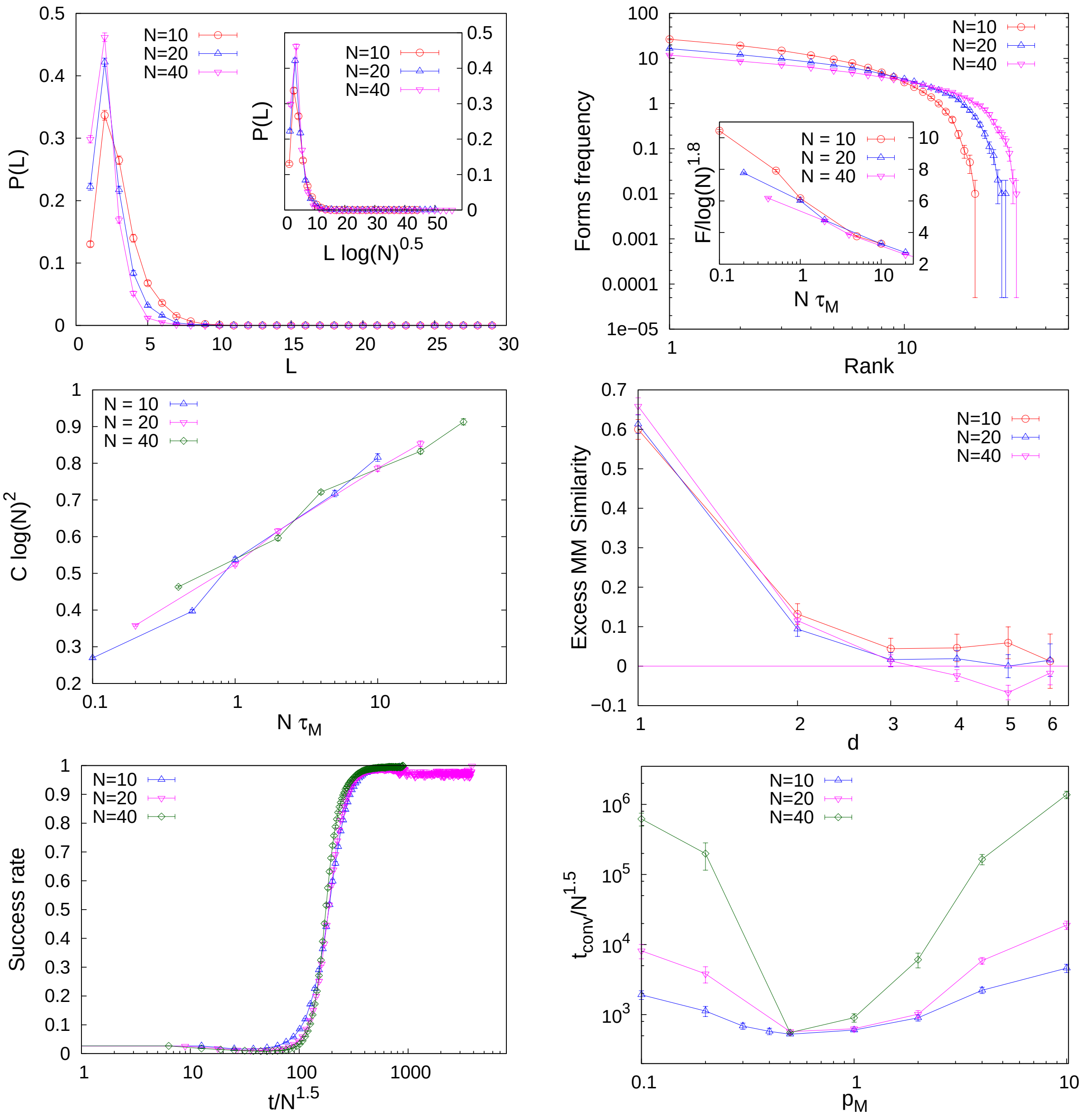

Figure S3. Dependence on N. Top Left. *Word length distribution.* In the main figure the distribution of word length L for different values of N, with $p_M = 1$ and $\tau_M = 1$ is reported. In the inset we report the same distribution with the word length rescaled by a factor $\log(N)^{0.5}$. The collapse in this case is very good, demonstrating an extremely weak dependence of the average word length on the population size N. **Top Right.** *Frequency-rank distribution for elementary forms.* In the main figure we show the frequency-rank distributions for elementary forms for different values of N, again with $p_M = 1$ and $\tau_M = 1$. We also report two fits obtained with a Yule (red solid line) $f(R) = aR^b c^R$, which has been hypothesized to reproduce the actual distribution in human languages [7], and a Yule-like (black solid line) distribution, $f(R) = \alpha \exp -\beta R^\gamma R^\delta$ [8]. The two functions coincide for $\gamma = 1$. The Yule function seems to fit better and better our distributions as M increases, while a finite-size distribution is better fitted by a Yule-like distribution. In the inset we show the number of distinct forms in the emerged lexicons as a function of τ_M, rescaled with the number N of agents, and for $p_M = 1$. The curves are again rescaled in order to overlap for high values of τ_M. **Center Left.** *Combinatoriality.* Combinatoriality C for different values of N as a function of τ_M, rescaled with the number N of agents, and for $p_M = 0.5$. **Center Right.** *Excess similarity of words as a function of the distance of the corresponding objects on the graph.* The difference between the actual similarity and its random value computed on a reshuffled communication system is shown (see the main text for details) for different values of N and for $\tau_M = 1$ and $p_M = 0.2$. Again, the results show a very weak dependence on N and the excess similarity becomes slightly more pronounced as N increases. **Bottom Left.** *Success rate as a function of time.* Success rate is reported as a function of time (number of games) for different values of N, with $p_M = 1$ and $\tau_M = 1$. The curves for different values of N collapse if time is rescaled with a factor $N^{1.5}$, as in the original Naming Game [9]. **Bottom Right.** *Convergence time.* Time needed for the system to reach convergence for different values of N, as a function of p_M and with $\tau_M = 1$. We rescaled the curves in order to superimpose the curves for the value of p_M where the convergence time is minimal.

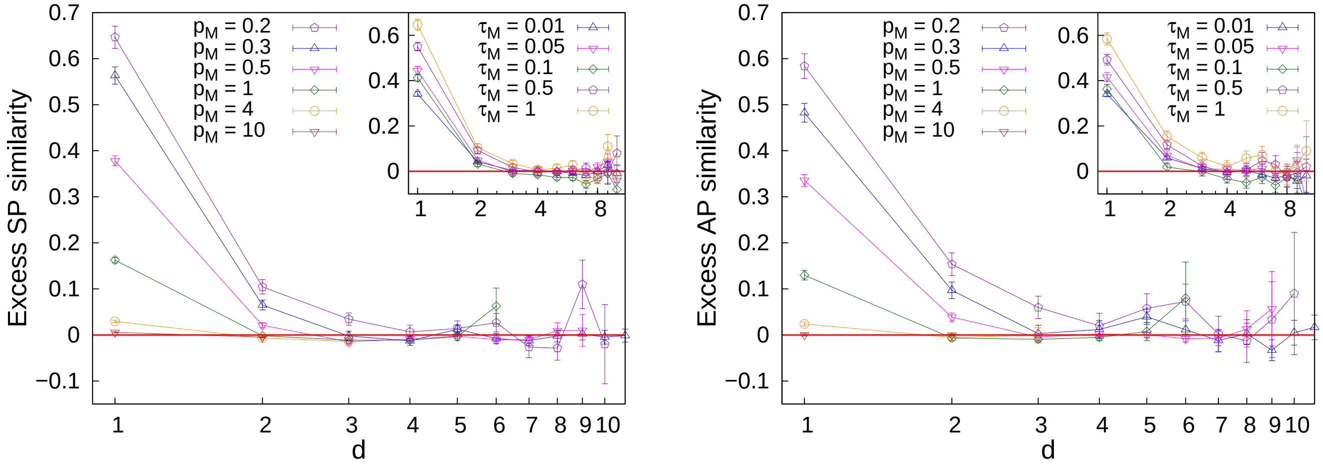

Figure S4. Left. *Excess SP similarity* The difference between the actual SP similarity and its random value computed on a reshuffled communication system is shown. The results are reported for $N = 10$ and $M = 100$. Results are shown for different values of the connecvity of the objects graph, keeping fixed $\tau_M = 1$ (main figure) and for different values of τ_M keeping fixed $p_M = 0.2$ (inset). **Right.** *Excess AP similarity* The difference between the actual AP similarity and its random value computed on a reshuffled communication system is shown. The results are reported for $N = 10$ and $M = 100$. Results are shown for different values of the connectivity of the objects graph, keeping fixed $\tau_M = 1$ (main figure) and for different values of τ_M keeping fixed $p_M = 0.2$ (inset).

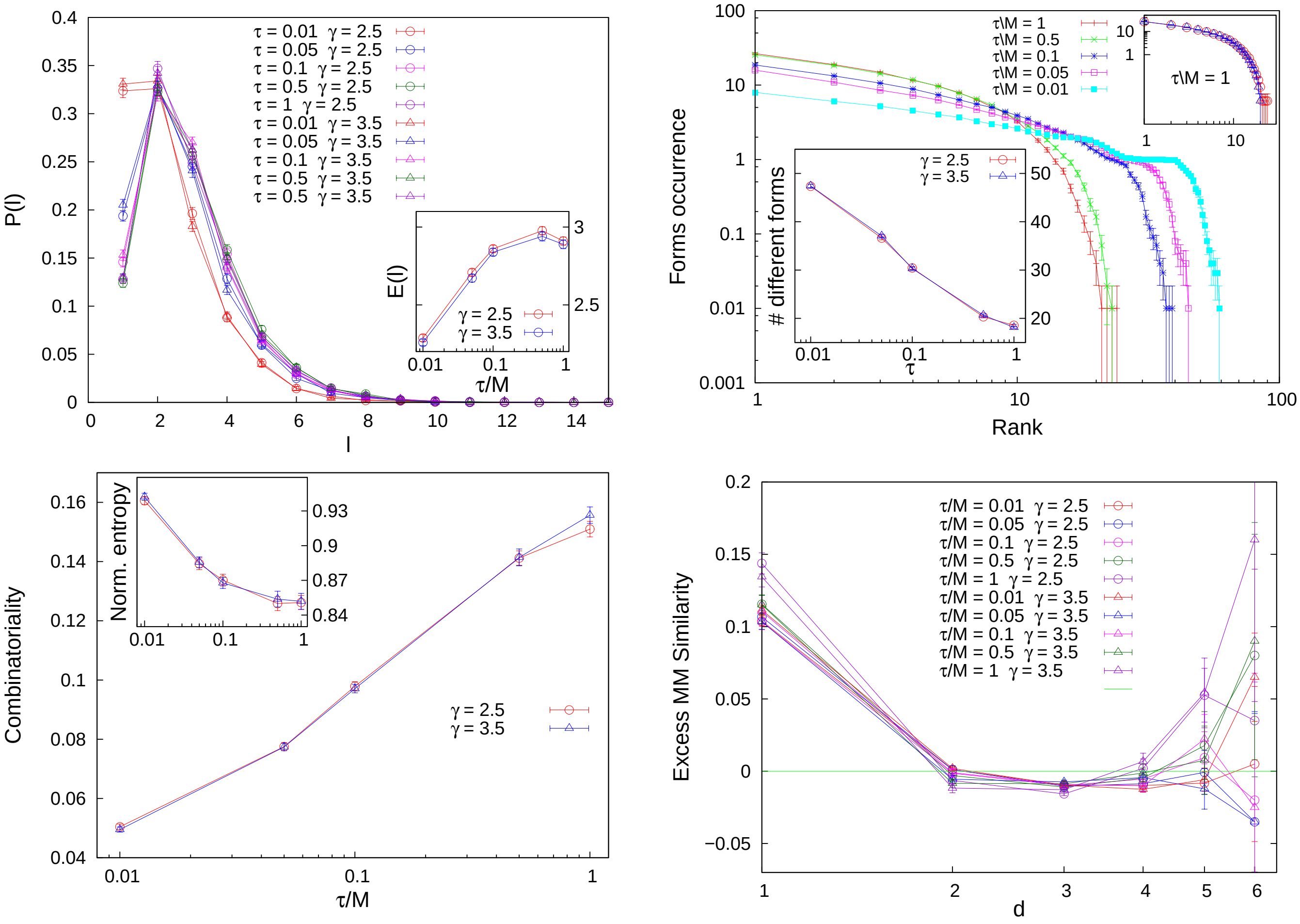

Figure S5. Dependence of the graph structure In this figure we report the results obtained by considering a different structure of the conceptual space. We considered in particular random scale-free networks obtained from the uncorrelated configuration model [6], characterized by a degree distribution $P(k) \sim k^{-\gamma}$. In our case we used $\gamma = 2.5$ and $\gamma = 3.5$. **Top Left.** *Word length distribution.* In the main figure the distribution of word length for different τ and for the two values of the degree distribution exponent γ is reported. The inset reports the *average word length* as a function of τ/M and for the two values of γ. As observed in the text, the word length distribution (and thus the average word length) does not depend on γ and is perfectly comparable to that obtained when considering Erdős -Rényi random graph (compare with fig. 2 in the main text). **Top Right.** *Frequency-rank distribution for elementary forms.* In the main figure the frequency-rank distribution for elementary forms is shown again for different values of the parameter τ and $\gamma = 2.5$. In the top inset we show the same distribution fixing $\tau/M = 1$ and for the two values of γ. In the bottom inset the *number of distinct elementary forms* composing the lexicon is reported, as a function of τ and for the two values of γ. Again the results do not depend on γ and are perfectly comparable to those obtained when considering Erdős -Rényi random graph. **Bottom Left.** *Combinatoriality.* Combinatoriality C (see the text for definition) for the two values of γ as a function of τ. In the inset the *normalized entropy*, as defined in the text, is reported, again for the two values of γ as a function of τ. **Bottom Right.** *Excess similarity of words as a function of the distance of the corresponding objects on the graph.* The excess MM similarity (see text) for different values of τ and for the two values of γ. As far as a non trivial structure of the world is preserved, the results do not depend on the actual value of the γ exponent. We here considered a graph with $M = 100$ nodes in order to have a greater variability. Note that the increase in excess similarity at high distances is an artifact, as the high error bars indicate, of the small number of objects at those distances in the graph. All the above results are averaged over 100 realizations of the process on the same graph with population size $N = 10$ and number of objects $M = 40$.
